# Supplementary material for: CDK12 Loss Promotes Prostate Cancer Development While Exposing Vulnerabilities to Paralog-Based Synthetic Lethality
Source: bioRxiv. 2024 Mar 21:2024.03.20.585990. Preprint. [Version 1] doi: 10.1101/2024.03.20.585990 (PMC10983964; doi:10.1101/2024.03.20.585990)
Supplement: Supplement 1 — Figure S1: Cdk12 is partially ablated in prostate epithelium by Probasin-driven Cre recombinase. Related to Figure 1. (A) Prostate epithelial Cdk12 ablation scheme. (B) CDK12 immunohistochemistry (IHC) and Cdk12 in situ hybridization (ISH) in 8-week-old WT and Cdk12pc−/− mice. (C) Percent epithelial cells immunonegative for CDK12 (Cdk12 KO cells) in prostate lobes of Cdk12pc−/− mice: anterior prostate (AP), ventral prostate (VP), dorsal prostate (DP), lateral prostate (LP). (n= 2–3 prostate cross sections from 6 mice). Figure S2: Cdk12 ablation in prostate epithelium of mixed background mice causes pre-cancerous lesions with aging. Related to Figure 1. (A) Hyperplasia with lost nuclear polarity and isonucleosis in prostate epithelium of 30-week-old mixed background Cdk12pc−/− mice. Note concentrated Ki67 staining in histologically abnormal regions. These regions are absent in wild-type (WT) controls. (B) Larger pre-cancerous lesions (indicated by dashed line) in prostate epithelia of 52-week-old mixed background Cdk12pc−/− mice. (C) Percent cross sectional area occupied by pre-cancerous lesions in prostate lobes of 52-week-old Cdk12pc−/− mice. Anterior prostate (AP), ventral prostate (VP), dorsal prostate (DP), lateral prostate (LP). (n= 2–3 prostate cross sections from each of 6–7 mice). *p<0.05, **p<0.01. Figure S3: Application of mT/mG model to isolate cells with active Cre recombinase and Cdk12 ablation; demonstration of abnormal morphology in organoids generated from Cdk12-null cells. Related to Figure 2. (A) Generation of a Pb-Cre;Cdk12f/f;mT/mG prostate mouse model to identify prostate epithelial cells with active Cre recombinase. (B) Basal cell isolation from Pb-Cre;Cdk12f/f;mT/mG prostate (52-week time point). (C) Cdk12 mRNA expression in 52-week Pb-Cre;Cdk12f/f;mT/mG prostate epithelial cells. BC, basal cells; LEC, luminal epithelial cells. (D) Organoids derived from Pb-Cre;Cdk12f/f;mT/mG prostate basal cells (52-week time point): Red/ Tom(+) contain intac [file media-1.pdf]

Figure S1

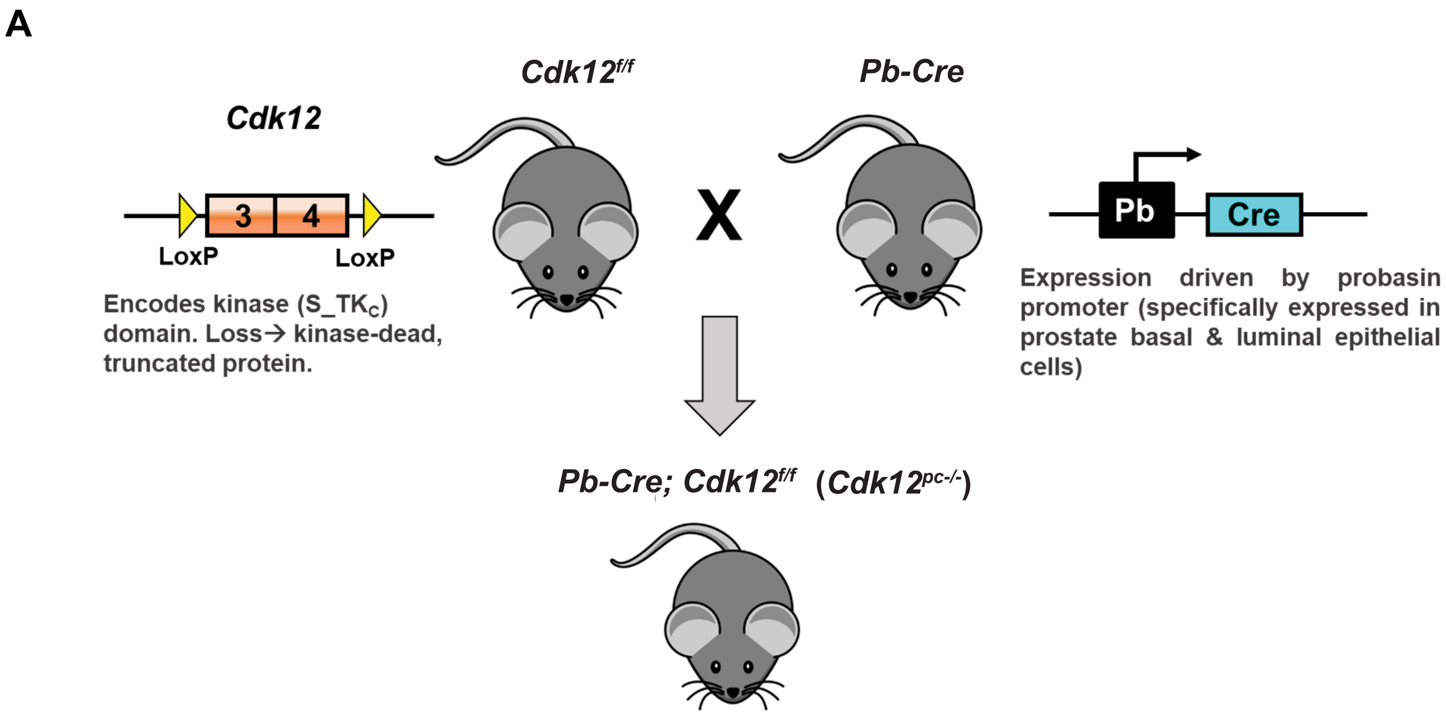

A
Cdk12
3
4
LoxP
LoxP
Encodes kinase (S_TKc) domain. Loss→ kinase-dead, truncated protein.
Cdk12f/f
X
Pb-Cre
Pb
Cre
Expression driven by probasin promoter (specifically expressed in prostate basal & luminal epithelial cells)
Pb-Cre; Cdk12f/f (Cdk12pc-/-)

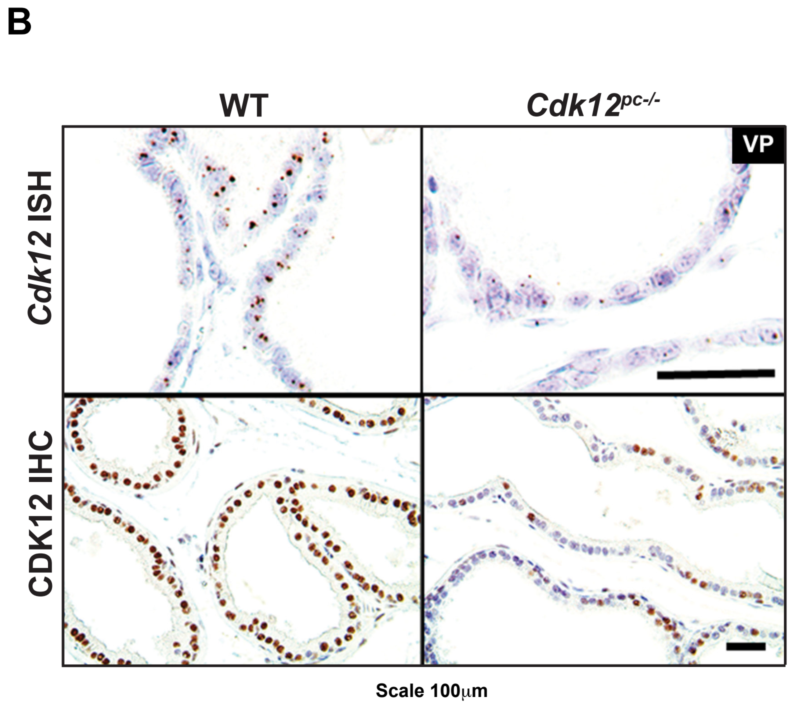

B
WT
Cdk12pc-/-
VP
Cdk12 ISH
CDK12 IHC
Scale 100μm

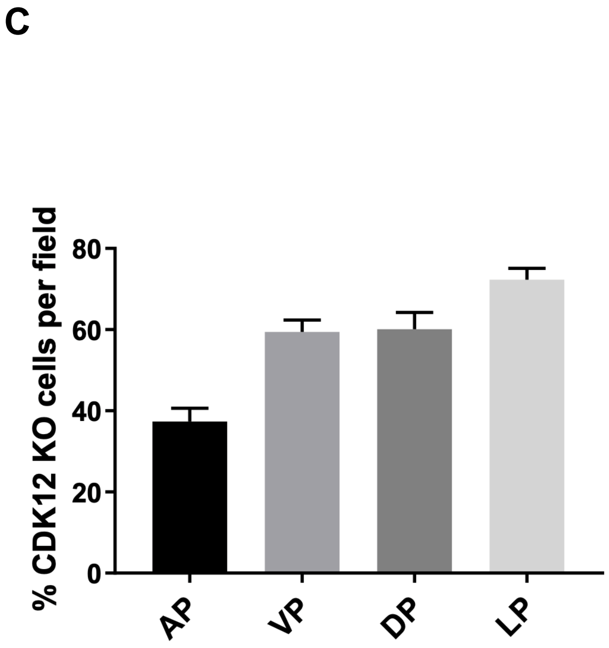

C
% CDK12 KO cells per field
0
20
40
60
80
AP
VP
DP
LP

Figure S2

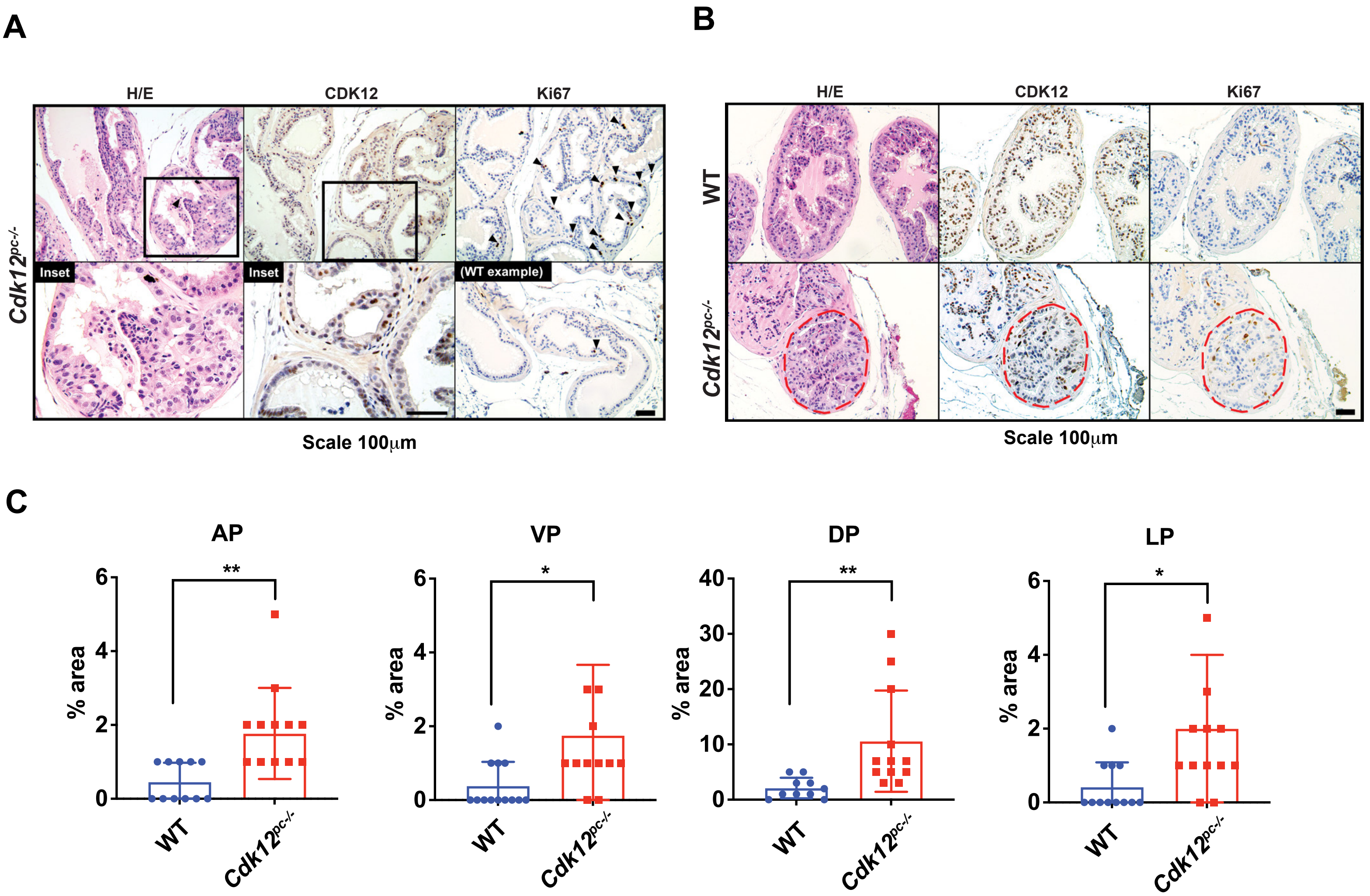
A
H/E
CDK12
Ki67
Cdk12pc-/-
Inset
Inset
(WT example)
Scale 100µm
B
H/E
CDK12
Ki67
WT
Cdk12pc-/-
Scale 100µm
C
AP
VP
DP
LP
% area
**
*
**
*
WT
Cdk12pc-/-

Figure S3

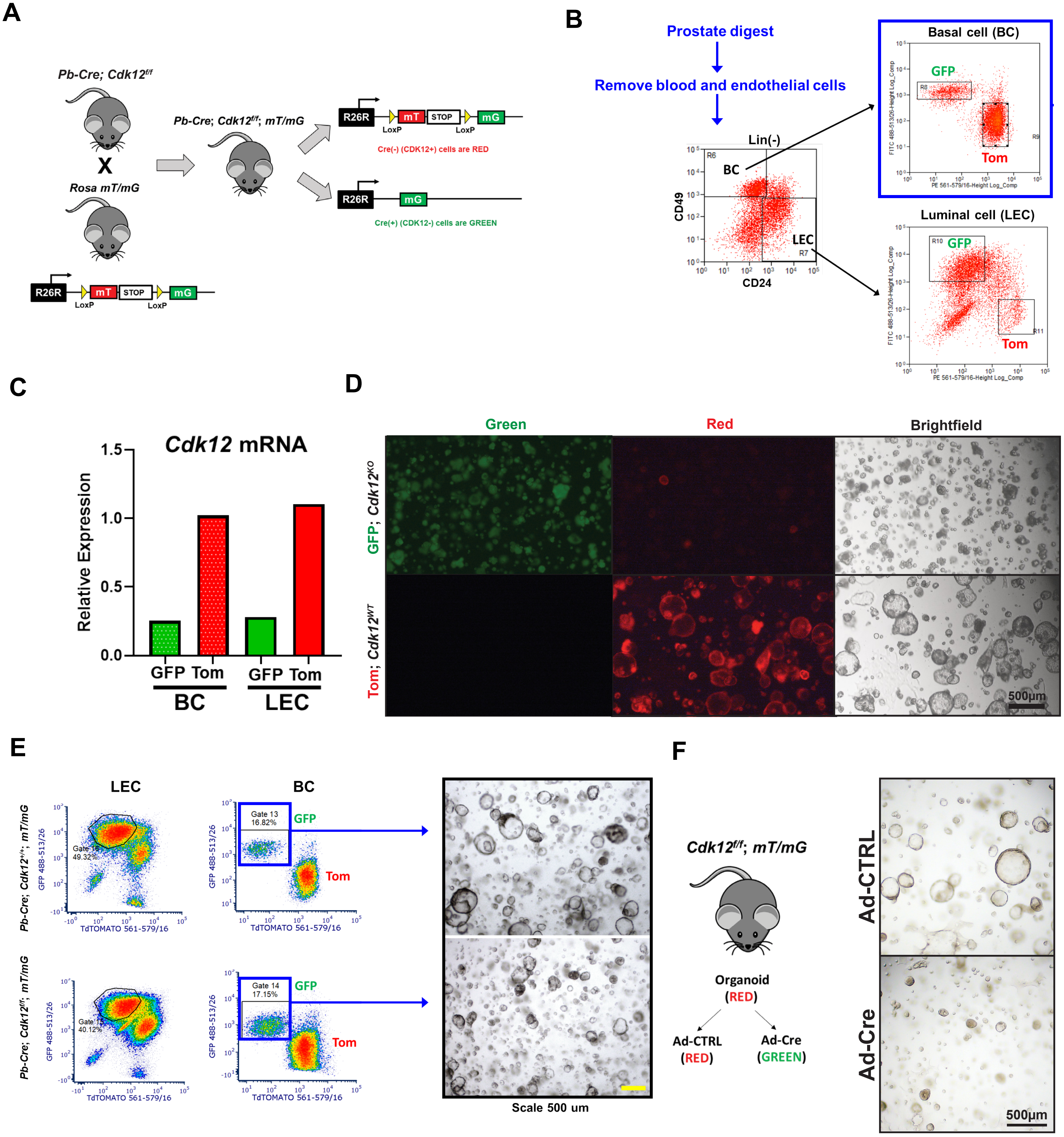

A
Pb-Cre; Cdk12f/f
X
Rosa mT/mG
Pb-Cre; Cdk12f/f; mT/mG
R26R
mT
STOP
mG
LoxP
Cre(-) (CDK12+) cells are RED
Cre(+) (CDK12-) cells are GREEN
B
Prostate digest
Remove blood and endothelial cells
Lin(-)
BC
LEC
CD49
CD24
Basal cell (BC)
Luminal cell (LEC)
GFP
Tom
C
Cdk12 mRNA
Relative Expression
GFP
Tom
BC
LEC
D
Green
Red
Brightfield
GFP; Cdk12KO
Tom; Cdk12WT
500µm
E
LEC
BC
Pb-Cre; Cdk12+/+; mT/mG
Pb-Cre; Cdk12f/f; mT/mG
Gate 13
16.82%
Gate 14
17.15%
Gate 16
49.32%
40.12%
TdTOMATO 561-579/16
GFP 488-513/26
Scale 500 um
F
Cdk12f/f; mT/mG
Organoid
(RED)
Ad-CTRL
(RED)
Ad-Cre
(GREEN)
Ad-CTRL
Ad-Cre
500µm

Figure S4

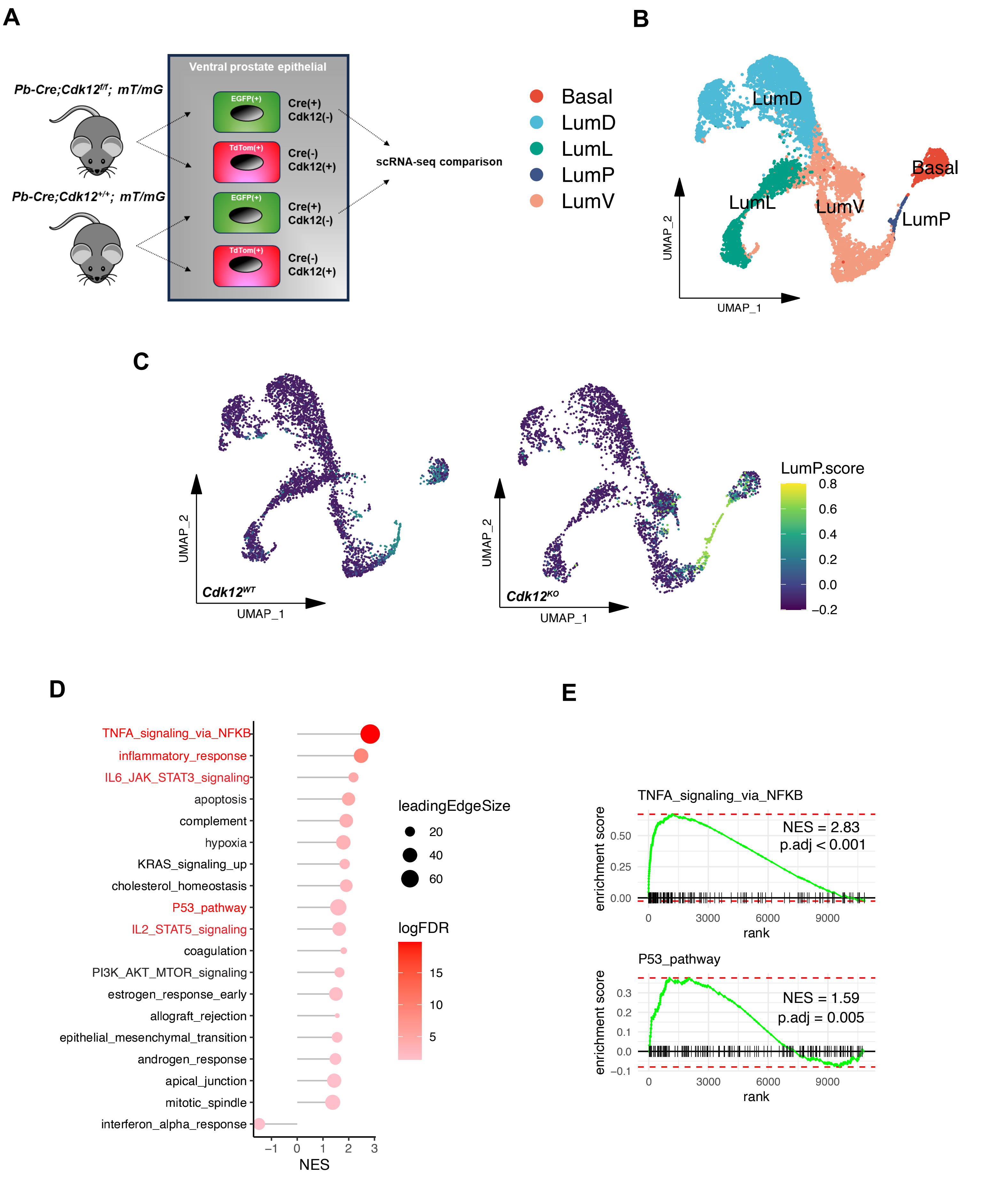
A
Pb-Cre;Cdk12f/f; mT/mG
Pb-Cre;Cdk12+/+; mT/mG
Ventral prostate epithelial
EGFP(+)
Cre(+) Cdk12(-)
TdTom(+)
Cre(-) Cdk12(+)
EGFP(+)
Cre(+) Cdk12(-)
TdTom(+)
Cre(-) Cdk12(+)
scRNA-seq comparison
B
Basal
LumD
LumL
LumP
LumV
UMAP_1
UMAP_2
C
Cdk12WT
Cdk12KO
UMAP_1
UMAP_2
LumP.score
0.8
0.6
0.4
0.2
0.0
−0.2
D
TNFA_signaling_via_NFKB
inflammatory_response
IL6_JAK_STAT3_signaling
apoptosis
complement
hypoxia
KRAS_signaling_up
cholesterol_homeostasis
P53_pathway
IL2_STAT5_signaling
coagulation
PI3K_AKT_MTOR_signaling
estrogen_response_early
allograft_rejection
epithelial_mesenchymal_transition
androgen_response
apical_junction
mitotic_spindle
interferon_alpha_response
−1 0 1 2 3
NES
leadingEdgeSize
20
40
60
logFDR
15
10
5
E
TNFA_signaling_via_NFKB
NES = 2.83
p.adj < 0.001
enrichment score
0.00 0.25 0.50
0 3000 6000 9000
rank
P53_pathway
NES = 1.59
p.adj = 0.005
enrichment score
−0.1 0.0 0.1 0.2 0.3
0 3000 6000 9000
rank

Figure S5

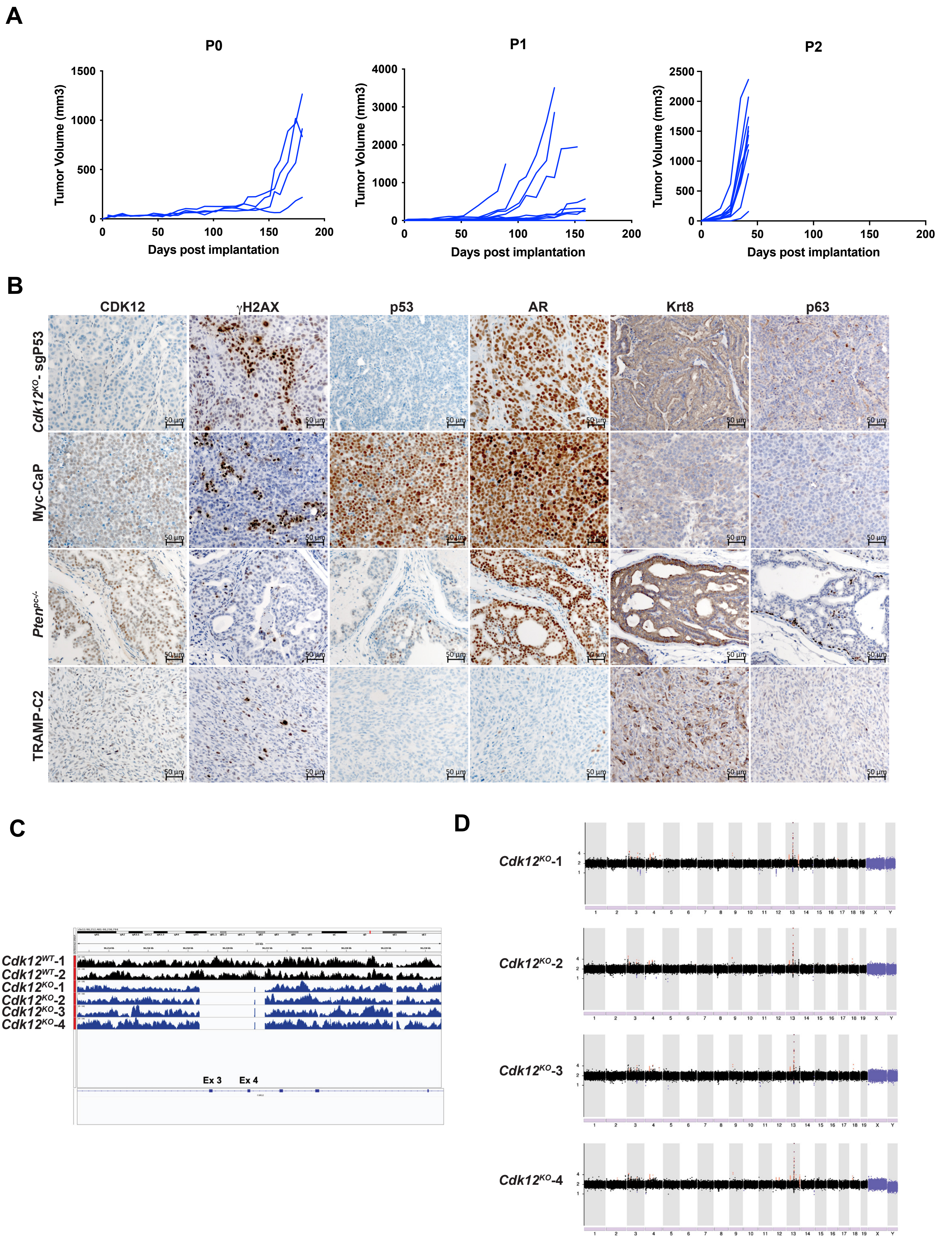

A
P0
P1
P2
Tumor Volume (mm3)
Days post implantation
0
50
100
150
200
500
1000
1500
2000
2500
3000
4000
B
CDK12
γH2AX
p53
AR
Krt8
p63
Cdk12KO- sgP53
Myc-CaP
Ptenpc-/-
TRAMP-C2
50 µm
C
Cdk12WT-1
Cdk12WT-2
Cdk12KO-1
Cdk12KO-2
Cdk12KO-3
Cdk12KO-4
Ex 3
Ex 4
D
Cdk12KO-1
Cdk12KO-2
Cdk12KO-3
Cdk12KO-4
1 2 3 4 5 6 7 8 9 10 11 12 13 14 15 16 17 18 19 X Y

Figure S6

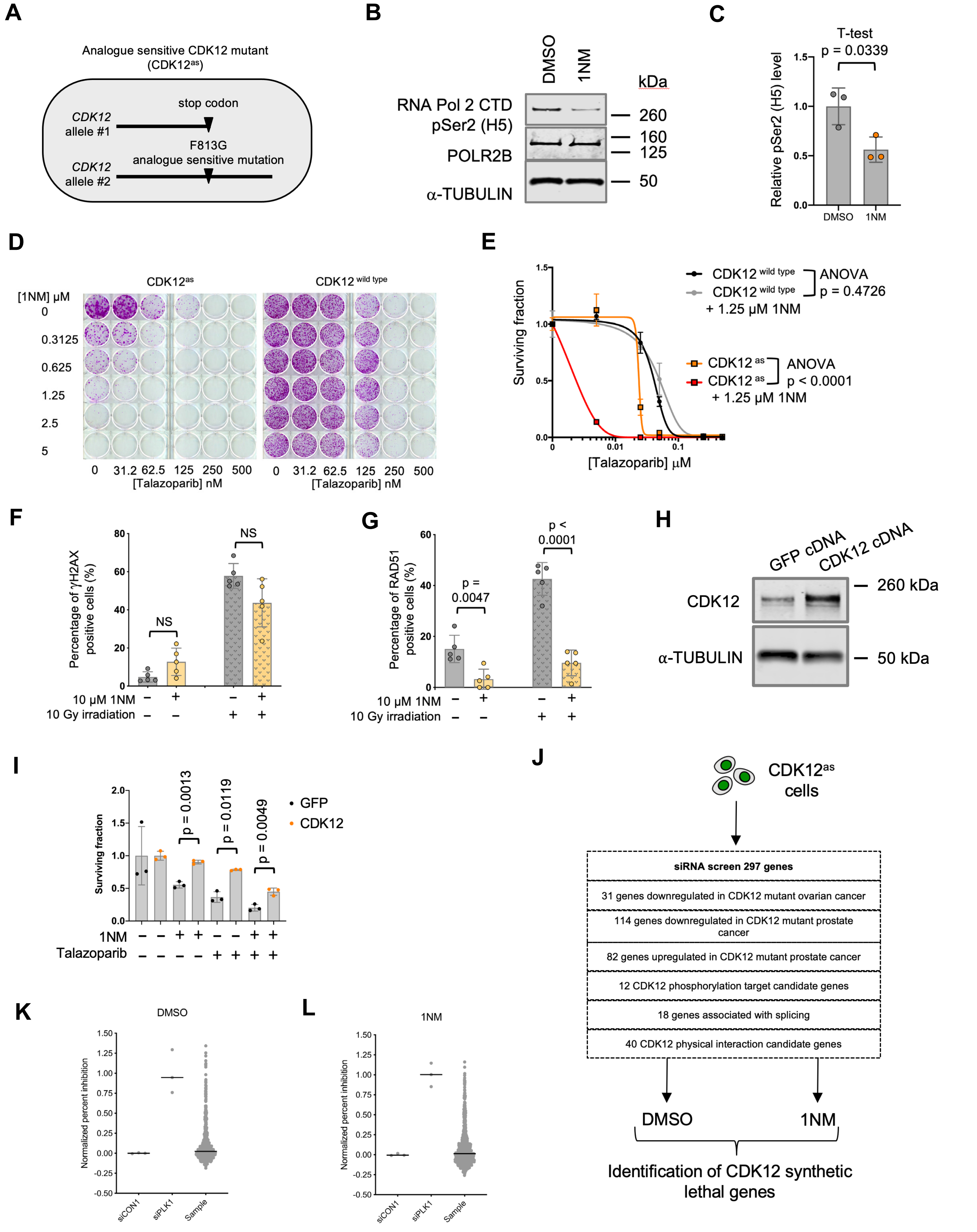
A
Analogue sensitive CDK12 mutant (CDK12as)
stop codon
CDK12 allele #1
F813G analogue sensitive mutation
CDK12 allele #2
B
DMSO
1NM
kDa
RNA Pol 2 CTD pSer2 (H5)
POLR2B
α-TUBULIN
260
160
125
50
C
T-test
p = 0.0339
Relative pSer2 (H5) level
DMSO
1NM
D
CDK12as
CDK12 wild type
[1NM] μM
0
0.3125
0.625
1.25
2.5
5
0 31.2 62.5 125 250 500
[Talazoparib] nM
E
Surviving fraction
[Talazoparib] μM
CDK12 wild type
CDK12 wild type + 1.25 μM 1NM
ANOVA p = 0.4726
CDK12 as
CDK12 as + 1.25 μM 1NM
ANOVA p < 0.0001
F
Percentage of γH2AX positive cells (%)
NS
NS
10 μM 1NM
10 Gy irradiation
G
Percentage of RAD51 positive cells (%)
p = 0.0047
p < 0.0001
10 μM 1NM
10 Gy irradiation
H
GFP cDNA
CDK12 cDNA
CDK12
α-TUBULIN
260 kDa
50 kDa
I
Surviving fraction
p = 0.0013
p = 0.0119
p = 0.0049
GFP
CDK12
1NM
Talazoparib
J
CDK12as cells
siRNA screen 297 genes
31 genes downregulated in CDK12 mutant ovarian cancer
114 genes downregulated in CDK12 mutant prostate cancer
82 genes upregulated in CDK12 mutant prostate cancer
12 CDK12 phosphorylation target candidate genes
18 genes associated with splicing
40 CDK12 physical interaction candidate genes
DMSO
1NM
Identification of CDK12 synthetic lethal genes
K
DMSO
Normalized percent inhibition
siCON1
siPLK1
Sample
L
1NM
Normalized percent inhibition
siCON1
siPLK1
Sample

Figure S7

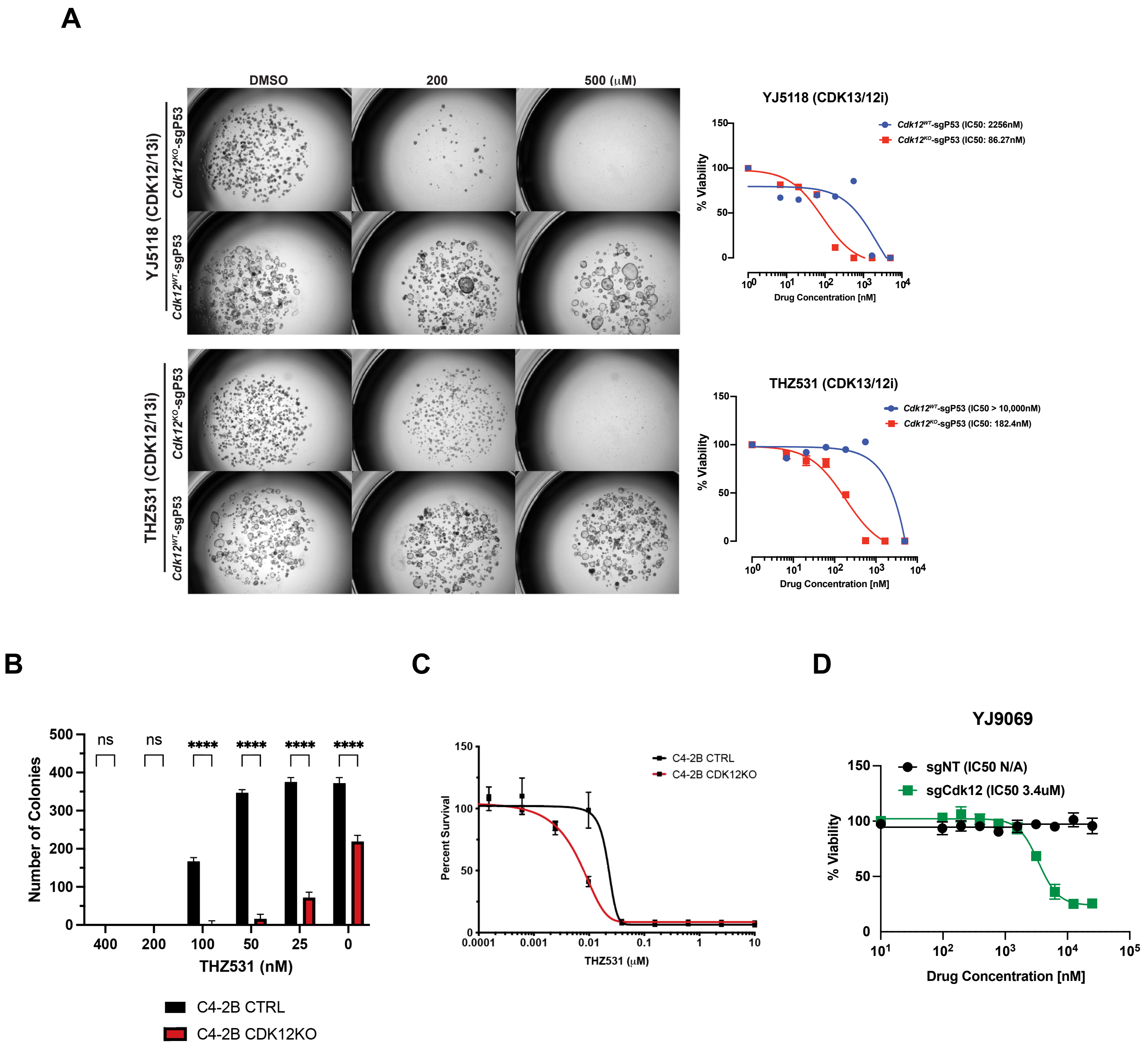
A
DMSO
200
500 (μM)
YJ5118 (CDK12/13i)
Cdk12KO-sgP53
Cdk12WT-sgP53
THZ531 (CDK12/13i)
Cdk12KO-sgP53
Cdk12WT-sgP53
YJ5118 (CDK13/12i)
Cdk12WT-sgP53 (IC50: 2256nM)
Cdk12KO-sgP53 (IC50: 86.27nM)
% Viability
Drug Concentration [nM]
THZ531 (CDK13/12i)
Cdk12WT-sgP53 (IC50 > 10,000nM)
Cdk12KO-sgP53 (IC50: 182.4nM)
% Viability
Drug Concentration [nM]
B
Number of Colonies
ns
ns
****
****
****
****
400
200
100
50
25
0
THZ531 (nM)
C4-2B CTRL
C4-2B CDK12KO
C
Percent Survival
C4-2B CTRL
C4-2B CDK12KO
THZ531 (μM)
D
YJ9069
sgNT (IC50 N/A)
sgCdk12 (IC50 3.4uM)
% Viability
Drug Concentration [nM]

Figure S8

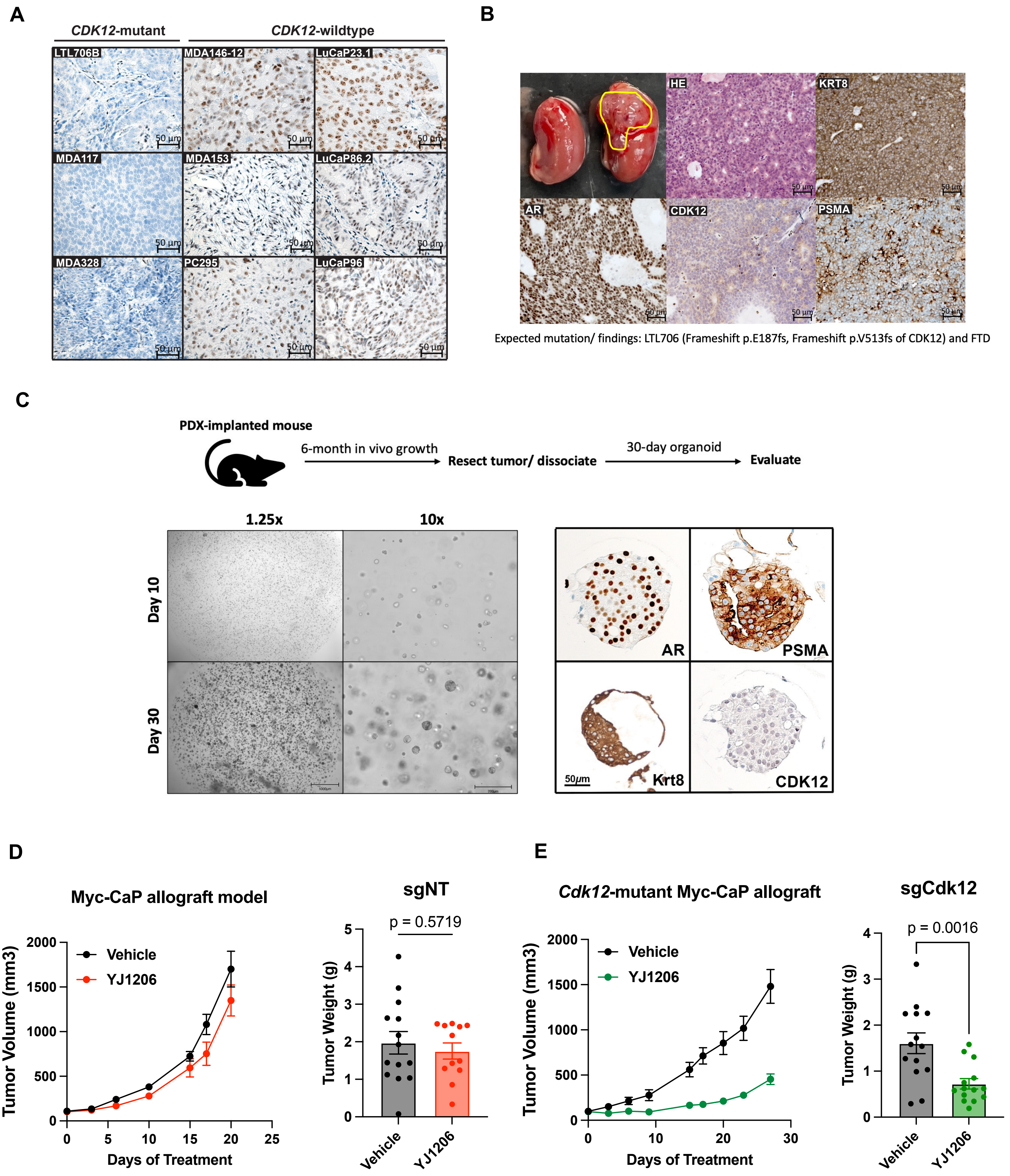

A
CDK12-mutant
CDK12-wildtype
LTL706B
MDA146-12
LuCaP23.1
MDA117
MDA153
LuCaP86.2
MDA328
PC295
LuCaP96
50 µm
B
HE
KRT8
AR
CDK12
PSMA
Expected mutation/ findings: LTL706 (Frameshift p.E187fs, Frameshift p.V513fs of CDK12) and FTD
C
PDX-implanted mouse
6-month in vivo growth
Resect tumor/ dissociate
30-day organoid
Evaluate
1.25x
10x
Day 10
Day 30
AR
PSMA
Krt8
CDK12
50µm
D
Myc-CaP allograft model
Vehicle
YJ1206
Tumor Volume (mm3)
Days of Treatment
sgNT
p = 0.5719
Tumor Weight (g)
E
Cdk12-mutant Myc-CaP allograft
sgCdk12
p = 0.0016
